# The genomic alterations in glioblastoma influence the levels of CSF metabolites

**DOI:** 10.1186/s40478-024-01722-1

**Published:** 2024-01-19

**Authors:** Daniel H. Wang, Yoko Fujita, Antonio Dono, Ana G. Rodriguez Armendariz, Mauli Shah, Nagireddy Putluri, Pavel S. Pichardo-Rojas, Chirag B. Patel, Jay-Jiguang Zhu, Jason T. Huse, Brittany C. Parker Kerrigan, Frederick F. Lang, Yoshua Esquenazi, Leomar Y. Ballester

**Affiliations:** 1 Department of Translational Molecular Pathology, The University of Texas MD Anderson Cancer Center, 2130 W. Holcombe Blvd., Suite 910, Houston, TX 77030 USA; 2 Vivian L. Smith Department of Neurosurgery, McGovern Medical School, The University of Texas Health Science Center at Houston, 6400 Fannin St., Suite 2800, Houston, TX 77030 USA; 3 Escuela de Medicina y Ciencias de la Salud, Tecnológico de Monterrey, Av. Ignacio Morones Prieto 3000, Sertoma, Monterrey, N.L 64710 Mexico; 4 Department of Pathology, The University of Texas MD Anderson Cancer Center, 1515 Holcombe Blvd, Houston, TX 77030 USA; 5 Advanced Technology Core, Baylor College of Medicine, 1 Baylor Plaza, Houston, TX 77030 USA; 6 Department of Molecular and Cellular Biology, Baylor College of Medicine, 1 Baylor Plaza, Houston, TX 77030 USA; 7 Department of Neuro-Oncology, The University of Texas MD Anderson Cancer Center, 1515 Holcombe Blvd., Unit 1002, BSRB S5.8116b, Houston, TX 77030 USA; 8 Memorial Hermann Hospital-Texas Medical Center, Houston, TX 77030 USA; 9 Department of Neurosurgery, The University of Texas MD Anderson Cancer Center, 1400 Holcombe Blvd., Room FC7.2000, Unit 442, Houston, TX 77030 USA; 10 Center for Precision Health, McGovern Medical School, The University of Texas Health Science Center at Houston, 7000 Fannin St., Suite 600, Houston, TX 77030 USA; 11 Neuropathology and Molecular Genetic Pathology, Department of Pathology, Department of Translational Molecular Pathology, 1515 Holcombe Blvd, Unit 85, Houston, TX 77030 USA

**Keywords:** Biomarker, CSF, Cerebrospinal fluid, Glioblastoma, Carnitine, Shikimate, GABA, Choline, Lactate, Metabolomics, *TP53*, *PTEN*

## Abstract

Cerebrospinal fluid (CSF) analysis is underutilized in patients with glioblastoma (GBM), partly due to a lack of studies demonstrating the clinical utility of CSF biomarkers. While some studies show the utility of CSF cell-free DNA analysis, studies analyzing CSF metabolites in patients with glioblastoma are limited. Diffuse gliomas have altered cellular metabolism. For example, mutations in isocitrate dehydrogenase enzymes (e.g., *IDH1* and *IDH2*) are common in diffuse gliomas and lead to increased levels of D-2-hydroxyglutarate in CSF. However, there is a poor understanding of changes CSF metabolites in GBM patients. In this study, we performed targeted metabolomic analysis of CSF from *n* = 31 patients with GBM and *n* = 13 individuals with non-neoplastic conditions (controls), by mass spectrometry. Hierarchical clustering and sparse partial least square-discriminant analysis (sPLS-DA) revealed differences in CSF metabolites between GBM and control CSF, including metabolites associated with fatty acid oxidation and the gut microbiome (i.e., carnitine, 2-methylbutyrylcarnitine, shikimate, aminobutanal, uridine, N-acetylputrescine, and farnesyl diphosphate). In addition, we identified differences in CSF metabolites in GBM patients based on the presence/absence of *TP53* or *PTEN* mutations, consistent with the idea that different mutations have different effects on tumor metabolism. In summary, our results increase the understanding of CSF metabolites in patients with diffuse gliomas and highlight several metabolites that could be informative biomarkers in patients with GBM.

**Supplementary Information:**

The online version contains supplementary material available at 10.1186/s40478-024-01722-1.

## Introduction

Glioblastoma, isocitrate dehydrogenase (IDH)-wildtype (GBM) is a highly aggressive brain tumor with poor prognosis and limited treatment options. A major challenge in the treatment of patients with GBM is the lack of reliable, non-invasive diagnostic and prognostic biomarkers for early detection, monitoring, and therapy selection. Cerebrospinal fluid (CSF) is in direct contact with the central nervous system (CNS) and CSF metabolites have the potential to reflect the metabolic alterations associated with tumor presence and progression [42, 46]. Analysis of CSF metabolites is less invasive than tissue biopsy and allows for serial collection at different timepoints over the course of disease. Yet, CSF biomarkers remain underutilized in patients with GBM and have not yet been incorporated into routine clinical practice due to technical challenges and the need for more studies to understand CSF metabolic changes.

Metabolomic analysis has revealed differences in metabolites between viable tumor, necrotic regions, and non-neoplastic regions in GBM tissue samples [12, 18]. Several studies have explored differences in plasma metabolites between GBM patients and controls [1, 14, 33, 38]. Machine learning analysis has also demonstrated changes in blood metabolites before surgery, after surgery, and following chemo-radiation in GBM patients [1]. Another study identified seven plasma molecules, associated with energy metabolism and signaling pathways catalyzing tumor proliferation and invasion, to be biomarkers that are elevated in GBM patients [14].

Although studies of CSF metabolites from GBM patients are limited, metabolomic analysis of CSF has shown promise in the characterization of CNS tumors. For example, differences in CSF metabolites between high-grade gliomas and healthy controls have been identified [34, 46]. Moreover, we have previously identified differences in CSF biomarkers in patients with diffuse gliomas based on *IDH1* mutation status, and in the CSF of patients with different types of CNS germ cell tumors [2, 17, 52]. Also, analysis of metabolites in CSF has been shown to predict malignant transformation and leptomeningeal metastasis in glioma patients [22].

In addition to the studies above, associating changes in CSF metabolites to brain tumors, studies suggest that gut microbiome-derived metabolites can influence the blood brain barrier [43, 50, 59]. Imbalances in the relative abundance of gut microbes (dysbiosis) can lead to disease in the CNS [3]. Studies have found differences in the gut microbiome of patients with multiple sclerosis (MS) versus healthy controls [10, 41]. In addition, gut dysbiosis have been described in the setting of brain tumors [13, 32]. In particular, alterations in gut levels of *Akkermansia sp.* have been associated with the presence of gliomas in humans and mice models, and metabolites of bacterial origin have been associated with neurotoxicity [41, 44].

In this study, we explored the metabolic profile of CSF in patients with GBM and investigated the correlation between CSF metabolites and genomic alterations. Our objective was to identify metabolic signatures in CSF that are associated with tumor presence, and to understand how these metabolites could serve as biomarkers for the diagnosis and monitoring of GBM patients.

## Methods

### Patients

**Fig. 1:**
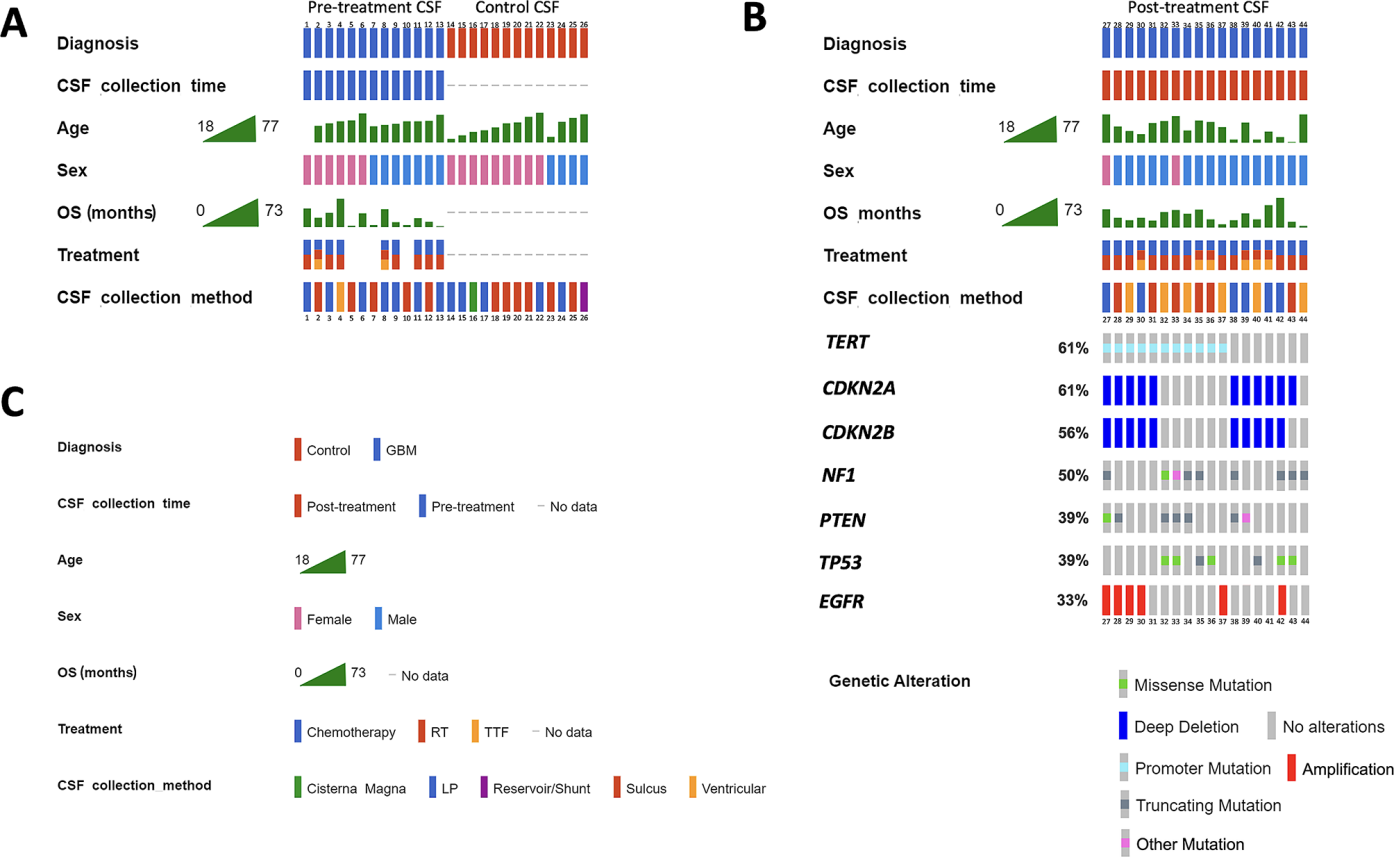
Patient characteristics. CSF samples were collected from patients with GBM (n = 31) and individuals without a history of cancer as controls (n = 13). (A) Characteristics of patients with CSF collected pre-treatment (n = 13) and controls (n = 13). Four patients (patients 5, 6, 7, and 10) received no additional treatment (i.e., no chemotherapy or radiation). (B) Characteristics of patients for which tumor mutation information was available (n = 18). All patients with mutation information had CSF collected post-treatment. (C) Legend for panels A and B. Overall survival (OS) spans from 2 to 73 months. Treatments included chemotherapy, radiation therapy (RT), tumor-treating field (TTF), or was not applicable for control patients. All GBM patients underwent surgery. CSF was collected via lumbar puncture (LP) (n = 18), sulcus sampling (n = 17), ventricular sampling (n = 7), reservoir/shunt (n = 1), or cisterna magna (n = 1)

CSF samples from 44 patients were included in the study: *n* = 31 from GBM patients and *n* = 13 from patients without a history of cancer (controls). Samples were collected via lumbar puncture (*n* = 18), sulcus sampling during resection (*n* = 17), ventricular catheter (*n* = 7), reservoir/shunt (*n* = 1), or cisterna magna (*n* = 1) (Fig. 1A).

### CSF processing

CSF samples were processed within 3 h of collection and centrifuged twice at 1,000 × *g* for 10 min at 4 °C. The cell pellet was discarded, and the supernatant was immediately stored at -80 °C until the time of analysis. 100 µL of each CSF sample were used for metabolomics analysis. CSF via lumbar puncture was collected utilizing the adult LP tray (Cat. 4306 C, CareFusion, IL, USA).

### Targeted metabolomics

Liquid chromatography-mass spectrometry (LC-MS) Single Reaction Monitoring (SRM) was used to characterize metabolites in CSF. Metabolites were measured using three different chromatographic methods. For each method, metabolites were normalized against the spiked internal standards and the data were log2-transformed (see Sup. Material 6 for details).

### Statistical analysis

The relative abundance of 125 metabolites was measured as part of a targeted metabolite profiling analysis. The resulting counts were normalized to an internal standard and log2-transformed. The data were additionally z-transformed for each metabolite across all samples for heatmap generation. Welch’s t-test was used for comparisons of metabolite levels between two groups. Kruskal-Wallis test with post hoc Dunn’s test for multiple comparisons was used for comparisons of metabolite levels between 3 groups. All analyses were performed using Python 3.8 and R Statistical Software (v4.2.2; R Core Team 2021). Significance calculations were performed using the Python library scikit-learn [45]. The University of Texas MD Anderson Cancer Center Department of Bioinformatics next-generation clustered heat map (NGCHM) software in R was used to generate heatmaps [6]. Sparse partial least squares-discriminant analysis (sPLS-DA) plots were generated using the mixOmics R toolkit [47]. Boxplots were generated using the R package ggpubr [28] and volcano plots using Matplotlib [21] in Python. Kaplan-Meier plots were generated using the kaplanmeier Python library [53], and cutoff values for determining high and low groups were found using the function *get.cutoff()* from the CutoffFinder R file [7].

### Mutation analysis

A subset of matched tumor samples was analyzed for genomic alterations by a targeted next-generation sequencing (NGS) panel interrogating 205 cancer-related genes for mutations and 26 genes for rearrangements (FoundationOne; Foundation Medicine, Inc.). This information was obtained from the patient’s electronical medical record.

## Results

### Patients and CSF samples

This study included CSF from 31 patients with IDH-wildtype glioblastoma (GBM), 23 men and 8 women, ranging in age from 18 to 76 years. Thirteen (13) patients had CSF samples collected pre-treatment, while 18 patients had CSF samples collected post-treatment (Fig. 1A, 1B). Somatic mutation analysis of tumor tissue was only available for the 18 patients with post-treatment CSF samples (Fig. 1B). In addition, CSF from 13 patients, (4 male and 9 female), ranging in age from 25 to 77 years, with non-neoplastic CNS diseases (i.e., hydrocephalus, stroke, aqueduct stenosis, migraine, arachnoid cyst, colloid cyst, and trauma) was used as control (Fig. 1A). The survival and event timeline of GBM patients in the study are illustrated in Sup. Material 1.

### Differences in metabolites between pre-treatment GBM and post-treatment GBM samples

**Fig. 2:**
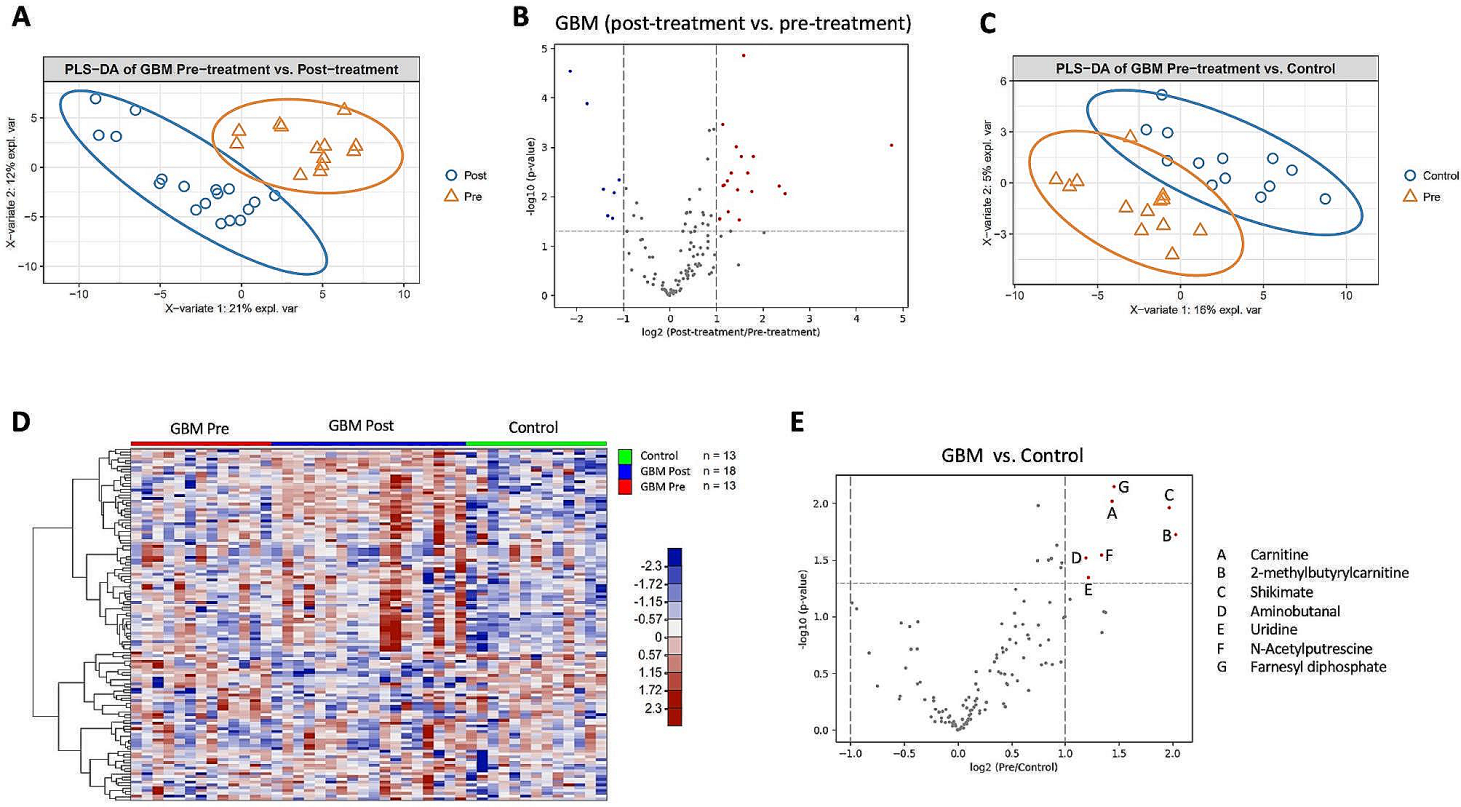
CSF metabolites differ between patients with GBM and controls. (**A**) sPLS-DA plot with the 31 GBM samples shows that GBM samples collected pre-treatment and post-treatment cluster in separate groups. (**B**) Volcano plot comparing CSF metabolites in CSF from GBM pre-treatment vs. GBM post-treatment. Colored points represent metabolites that are present at significantly different levels and fold change (-log10(p-value) > 1.3, log2(Post/Pre) > + 1 or < -1) in CSF samples from GBM pre-treatment vs. GBM post-treatment. Several CSF metabolites are present at significantly different levels in GBM pre-treatment compared to GBM post-treatment. P-value and fold change for differentially abundant metabolites are listed in Supplementary Material 5. (**C**) sPLS-DA plot with the GBM pre-treatment (*n* = 13) and control (*n* = 13) CSF samples shows that GBM and control samples cluster in separate groups. (**D**) Supervised heat map of metabolite levels (*n* = 125) in CSF samples from patients with GBM (*n* = 31) and controls (*n* = 13). GBM patients either had CSF collected pre-treatment (*n* = 13) or post-treatment (*n* = 18). (**E**) Volcano plot comparing CSF metabolites in CSF from GBM vs. controls. Colored points represent metabolites that are present at significantly different levels and fold change (-log10(p-value) > 1.3, log2(GBM/Control) > + 1 or < -1) between GBM and control CSF samples. Seven metabolites are present at significantly higher levels in the CSF of GBM patients

125 metabolites were identified in CSF as part of the targeted metabolic analysis. Statistical comparisons of metabolite levels were performed between GBM pre-treatment (GBM pre-tx) and control patients, as well as between GBM pre-treatment and post-treatment samples. sPLS-DA demonstrated differences between pre-treatment and control, as well as between pre-treatment and post-treatment GBM CSF samples (Fig. 2A), (Fig. 2B). Therefore, pre- and post-treatment CSF samples were analyzed separately.

### Differences in CSF metabolites based on sex

There is a difference in the proportion of male and female patients between the pre- and post-treatment GBM cohorts, with a near-even split (46% female) in the pre-treatment cohort and disproportionately more male patients (11% female) in the post-treatment cohort. To confirm that the metabolic differences between pre- and post-treatment CSF samples are not due to differences in the proportion of female patients, we performed significance and fold change calculations on the GBM cohort based on sex. We identified a small number of metabolites (2-methylbutyrylcarnitine, methyl methoxyacetate, 2-hydroxyglutarate, cytosine, cholesterol sulfate, methylparaben) that are present at different levels between male and female patients in the GBM cohort (pre- and post-treatment samples) (Sup. Material 2A). Similarly, we identified a small number of metabolites (2-methylbutyrylcarnitine, 2-hydroxyglutarate, allantoin, glycine) that are different between male and female patients in the pre-treatment GBM cohort (Sup. Material 2B). To exclude variability due to patient’s sex, we compared pre-treatment and post-treatment CSF from male patients only, and we identified several metabolites that are significantly different between male pre- and post-treatment GBM CSF (3-Hydroxy-3-methylglutaric acid, stearic acid, uridine, spaglumic acid, hydroxyisocaproic acid, myristoleic acid, methyl hippurate, aminobutanal, alanine, kynurenine, s-ribosyl-L-homocysteine, alpha-ketoglutarate) (Sup. Material 2C). Therefore, the differences in CSF metabolites between pre-treatment and post-treatment GBM samples are not due only to sex.

### Differences in metabolites between pre-treatment GBM and control CSF samples

**Fig. 3:**
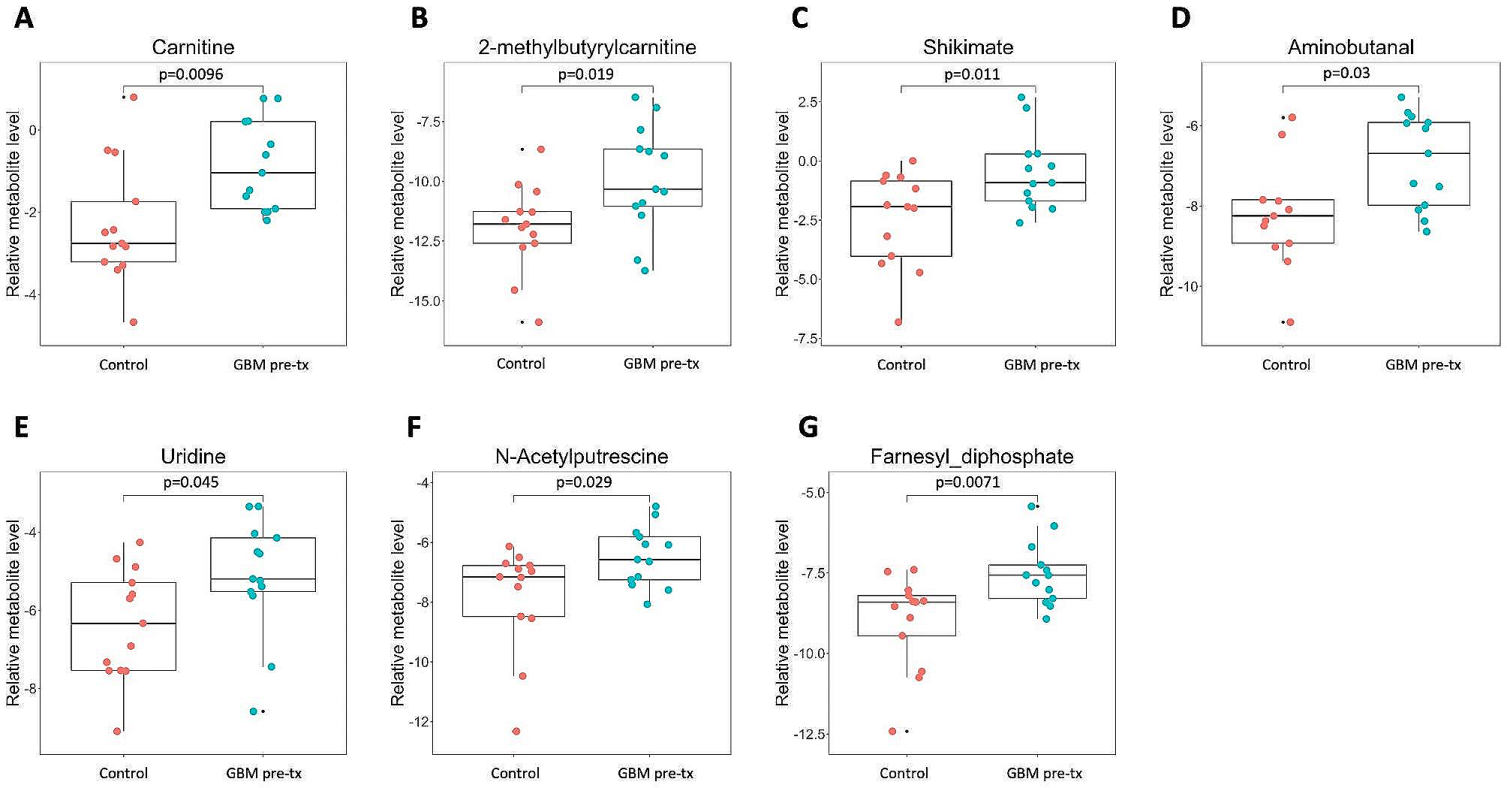
CSF from GBM patients exhibits significantly higher levels of (**A**) carnitine, (**B**) 2-methylbutyrylcarnitine, (**C**) shikimate, (**D**) aminobutanal, € uridine, (**F**) N-acetylputrescine, and (**G**) farnesyl diphosphate than control CSF. Each colored dot represents a patient, small black dot indicates samples that are 1.5 times the interquartile range above the upper quartile or below the lower quartile

sPLS-DA demonstrated differences in CSF metabolites between GBM (pre-treatment) and control samples (Fig. 2C). Supervised hierarchical clustering based on the squared Euclidean distance (Ward’s method) revealed differences in overall metabolite abundance between GBM pre-tx and controls (Fig. 2D). Volcano plots illustrated fold-change and probability values for each identified metabolite and highlighted differentially abundant metabolites. Several metabolites showed statistically significantly different levels between GBM pre-tx and control CSF (Fig. 2E). In particular, carnitine, 2-methylbutyrylcarnitine, shikimate, aminobutanal, uridine, N-acetylputrescine, and farnesyl diphosphate were present at significantly higher levels in the CSF of GBM pre-tx patients compared to controls (Fig. 3).

### Correlation between CSF metabolite levels and somatic mutations in GBM

**Fig. 4:**
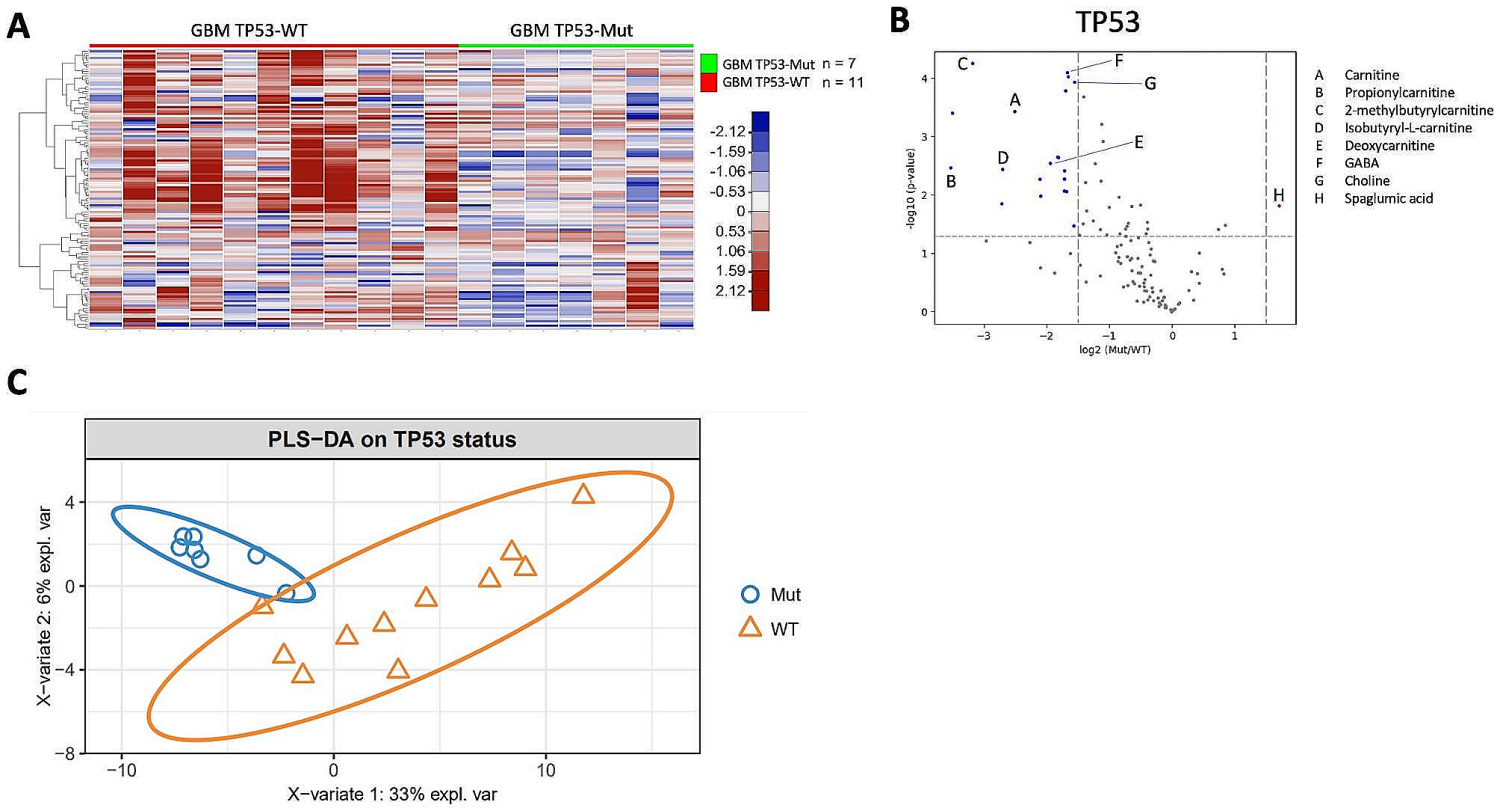
CSF metabolite levels differ with *TP53* mutation status. (**A**) Supervised heat map of metabolites (*n* = 125) with CSF samples grouped based on *TP53* status (mutant vs. wildtype). (**B**) Volcano plot of metabolites comparing GBM-*TP53*-mutant vs. GBM-*TP53*-wildtype. Colored points represent metabolites that are present at significantly different levels (-log10(p-value) > 1.3, log2(Mut/WT) > + 1.5 or < -1.5) between GBM-*TP53*-wildtype and GBM-*TP53*-mutant samples. Five carnitine compounds, choline, and γ-aminobutyric acid (GABA) are highly abundant in CSF from *TP53*-wildtype patients. (**C)** sPLS-DA plot of CSF metabolites status shows clear separation between *TP53*-mutant vs. *TP53*-wildtype samples

**Fig. 5:**
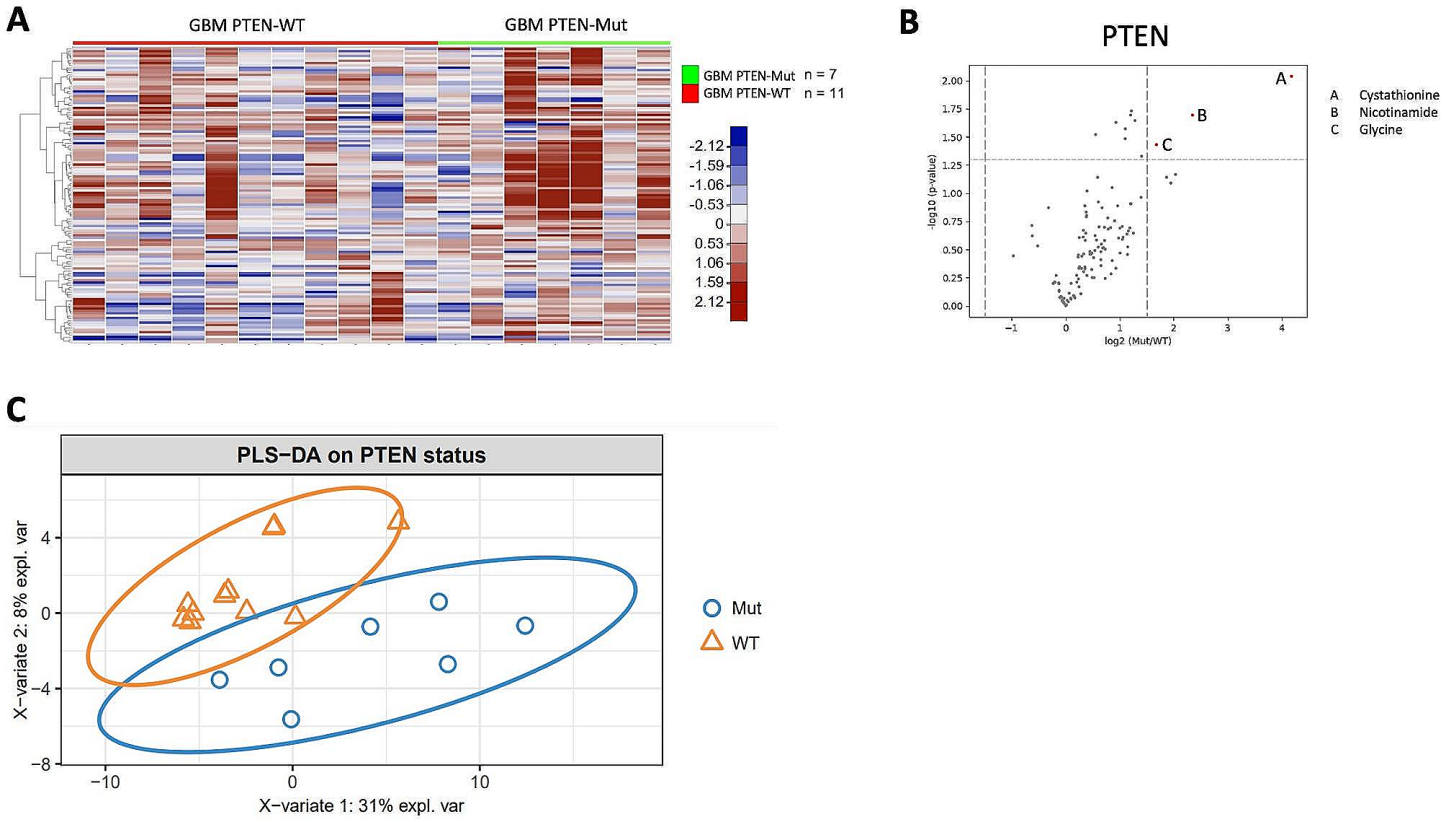
CSF metabolite levels differ with *PTEN* mutation status. (**A**) Supervised heat map based on *PTEN* status (mutant vs. wildtype). (**B**) Volcano plot of metabolites comparing CSF samples from patients with GBM-*PTEN*-mutant vs. GBM-*PTEN*-wildtype. (**C**) sPLS-DA plot shows clear separation between GBM-*PTEN*-mutant and GBM-*PTEN*-wildtype CSF samples

Somatic mutation analysis of tumor tissue was available for the 18 GBM patients with CSF collected post-treatment (Fig. 1B). The most common mutations identified were *TERT* promoter (*TERTp*), *CDKN2A/B, NF1, PTEN, TP53*, and *EGFR*. Global analysis of metabolites shows a statistically significant difference in CSF metabolite levels between patients with *TP53*-wildtype and *TP53*-mutant GBM (Fig. 4), as well as *PTEN*-mutant and *PTEN*-wildtype GBM (Fig. 5). Only minor significant differences in metabolites were identified according to *TERTp, CDKN2A/B, NF1 or EGFR* mutation status.

**Fig. 6:**
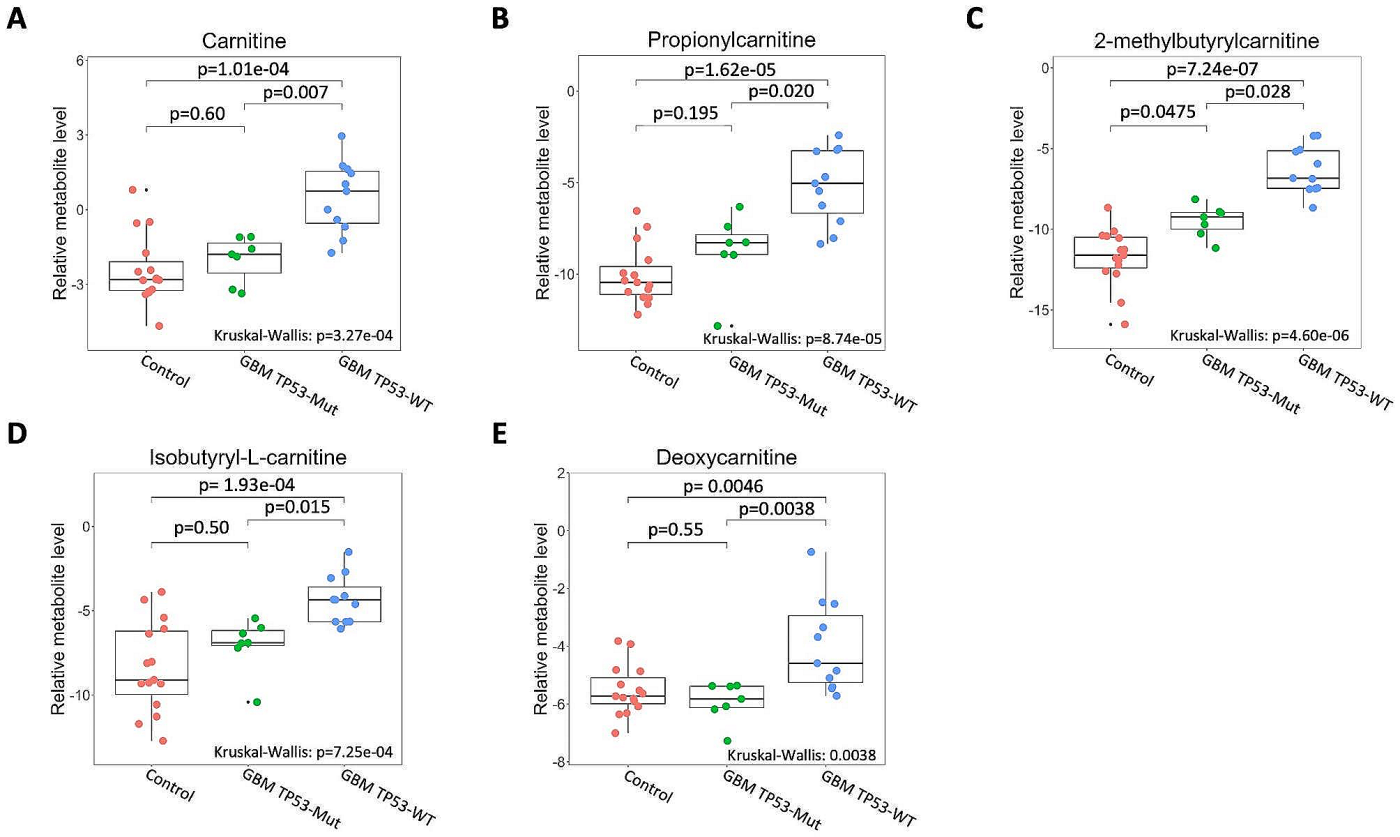
Pairwise comparisons of carnitine compound levels in CSF of control, GBM-*TP53*-mutant, and GBM-*TP53*-wildtype patients. (**A**) carnitine, (**B**) propionylcarnitine, (**C**) 2-methylbutyrylcarnitine, (**D**) isobutyryl-L-carnitine, and (**E**) deoxycarnitine. The abundance of carnitine compounds was not significantly different between control and GBM-*TP53-*mutant groups except for 2-methylbutyrylcarnitine (**C**). Metabolite abundance was significantly different between control and GBM-*TP53-*wildtype samples for all 5 carnitine compounds

Analysis of the levels of carnitine compounds revealed lower levels of these metabolites (i.e., carnitine, propionylcarnitine, 2-methylbutyrylcarnitine, isobutyryl-L-carnitine, and deoxycarnitine) in CSF from patients with *TP53-*mutant versus *TP53*-wildtype GBM (Fig. 6). When compared to the control CSF, *TP53*-mutant samples did not show significantly different levels for four of the five carnitine compounds (exception: 2-methylbutyrylcarnitine). In contrast, the level of all carnitine compounds in the CSF from patients with *TP53*-wildtype GBM was significantly higher across the board, compared to that in CSF from control patients or *TP53*-mutant GBM patients (Fig. 6).

**Fig. 7:**
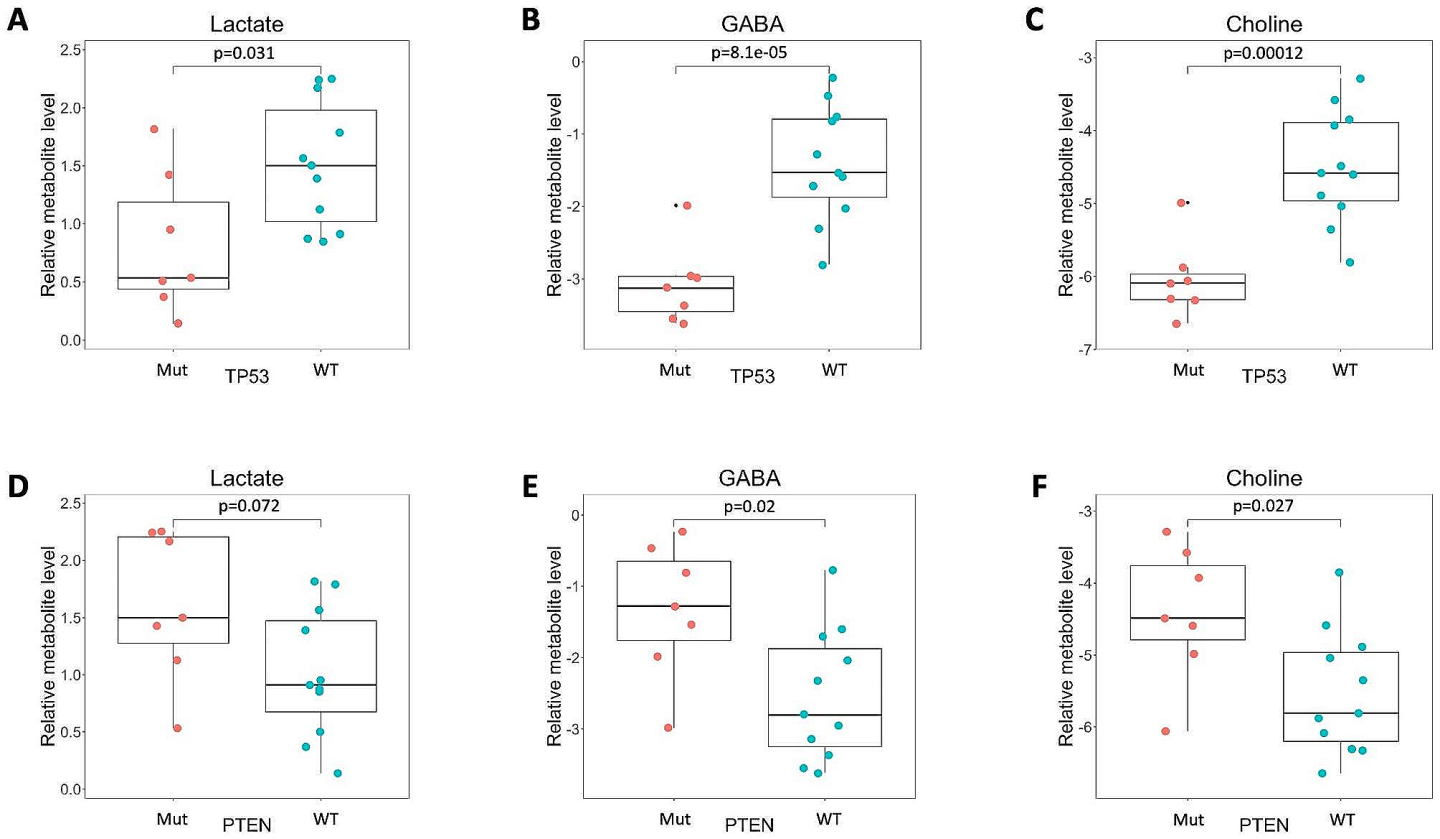
Levels of metabolites measured by MRS (Lactate, γ-aminobutyric acid (GABA), and choline) are influenced by tumor mutations. CSF samples from GBM *TP53*-wildtype patients show significantly elevated levels of (**A**) lactate, (**B**) GABA, and (**C**) choline. CSF samples from GBM *PTEN*-mutant patients show elevated levels of (**D**) GABA, (**E**) lactate, and (**F**) choline. Each colored dot represents a patient, small black dot indicates samples that are 1.5 times the interquartile range above the upper quartile or below the lower quartile

We next analyzed the levels of clinically-relevant metabolites that are routinely measured by magnetic resonance spectroscopy (MRS) in patients with GBM, including lactate, γ-aminobutyric acid (GABA), and choline. The data showed significantly lower levels of lactate, GABA and choline in CSF from patients with *TP53*-mutant GBM compared to *TP53*-wildtype. The data also showed higher levels of lactate, GABA, and choline in the CSF of patients with *PTEN*-mutant GBM compared to *PTEN*-wildtype GBM (Fig. 7).

### Correlation between CSF metabolites and survival in patients with GBM

**Fig. 8:**
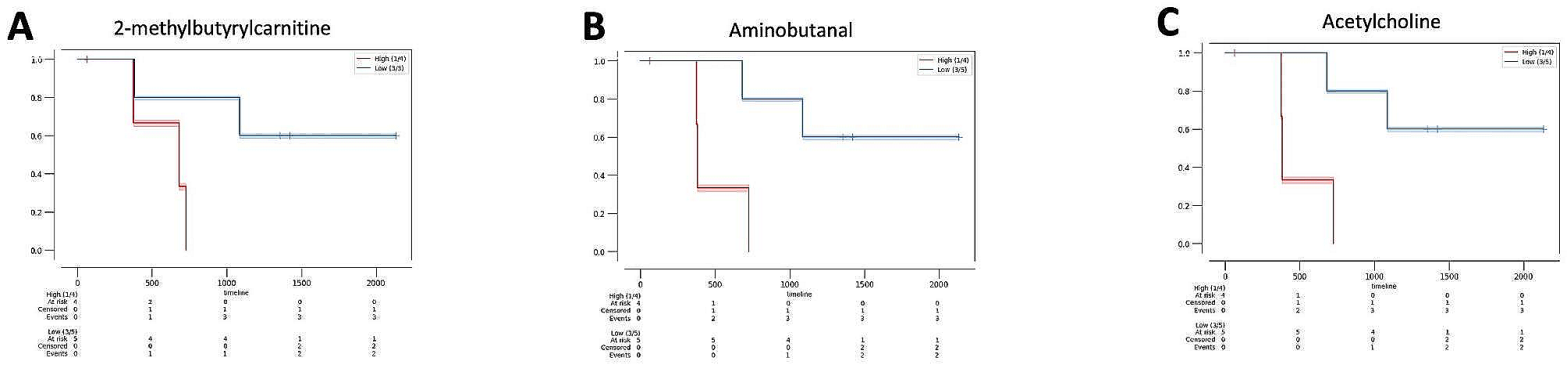
Kaplan-Meier plots showing survival probability for 9 GBM, IDH-wildtype patients divided into “high” (in red) and “low” (in blue) metabolite level groups. A patient is classified as “high” if the individual metabolite level is above the cutoff value calculated by CutoffFinder and classified as “low” if below the cutoff. CSF levels of 2-methylbutyrylcarnitine, aminobutanal, and acetylcholine are all inversely associated with survival. (**A**) 2-methylbutyrylcarnitine (*p* = 0.046); median survival in days (High = 681, Low = N/A). (**B**) Aminobutanal (*p* = 0.022); median survival in days (High = 382, Low = N/A). (**C**) Acetylcholine (*p* = 0.022); median survival in days (High = 382, Low = N/A)

We analyzed the correlation between metabolite levels in CSF and overall survival (OS) for the 9 pre-treatment GBM samples (Sup. Material 4). To evaluate if metabolites levels correlate with overall survival, we separated patients into “high” or “low” groups for each metabolite based on the cutoff value calculated from CutoffFinder’s fit of mixture model [7]. Kaplan-Meier plots were generated for each metabolite. OS was compared between patients with “high” or “low” levels for each of the 125 metabolites measured in the study. Three metabolites showed a statistically significant association with OS in patients with GBM (2-methylbutyrylcarnitine, aminobutanal, and acetylcholine) (Fig. 8).

### Metabolites and age

We performed a linear regression analysis between age and metabolite levels, for each metabolite, for the entire cohort of glioblastoma samples (pre- and post-treatment) and no significant correlation was observed (R^2 value ranged from 0.15 to 0.000055). We performed the same analysis with the pre-treatment samples and did not observe a significant correlation between age and metabolite levels for any of the metabolites analyzed (R^2 value ranged from 0.52 to 8.12E-07). We also performed the same analysis with the post-treatment cohort and did not observe a significant correlation between age and metabolite levels for any of the metabolites analyzed (R^2 value ranged from 0.30 to 1.76E-06).

## Discussion

Our results in this study and previous studies demonstrate differences in CSF metabolites between patients with diffuse gliomas and individuals without brain cancer [2, 22, 34]. Differences in levels of CSF metabolites may be due to the metabolic activity of cancer cells and/or the effect of tumor cells on overall brain activity [40, 42]. In this study, we found several metabolites that are present at significantly different levels in CSF of patients with GBM compared to controls. The data also show that GBM mutations may influence the levels of metabolites in CSF, including metabolites that are routinely analyzed by MRS with advanced brain MRI scans. Moreover, our current and previous data show that a metabolite considered to be of bacterial origin is significantly altered in the CSF of patients with GBM [2].

### Carnitine association between GBM and lipid metabolism

Carnitine and 2-methylbutyrylcarnitine were found to be present at significantly higher levels in the CSF of GBM patients compared to controls (Fig. 3A, 3B). GBM cells have been shown to exhibit elevated levels of carnitine [15], and although this was found in our study, it is unclear what role the GBM size, GBM location, and other factors may play. Carnitine and acyl-carnitines are crucial components of the carnitine shuttle system (CSS), which facilitates the transport of fatty acids into the mitochondria for fatty acid oxidation (FAO) (Sup. Material 3A) [8, 20, 35]. Recent studies have implicated FAO as an important energy production pathway in GBM as well as a key mediator of GBM plasticity and adaptability [26, 31, 49]. An increase in the CSF levels of carnitine found in this study may indicate increased reliance on lipid metabolism in GBM. Organic cation transporter OCTN2 (SLC22A5) is the primary transporter of dietary carnitine throughout the body, and OCTN2 is highly expressed in the brain of GBM patients compared to healthy brains [15, 24, 29]. Consequently, the higher carnitine levels observed in the CSF of the GBM patients evaluated here may be facilitated by upregulation of OCTN2, which in turn could potentially be a response to greater demand for carnitine by enhanced fatty acid oxidation in GBM.

### Shikimate levels and the gut microbiome

In our previous study of CSF metabolites, we found an association between higher CSF levels of shikimate and *IDH*-wildtype gliomas compared to controls [2], a finding recapitulated in Fig. 3C. CSF shikimate levels have also been found to be elevated in patients with autism spectrum disorder [5]. Shikimate is an intermediate in the shikimate pathway. This pathway is not present in animals but it is used by plants, bacteria, algae, and other microorganisms in the biosynthesis of aromatic compounds (Sup. Material 3B) [19]. While the shikimate pathway is active in some bacteria present in the human gut microbiome, transcriptome analysis shows that most gut bacteria do not possess a complete shikimate pathway [36]. However, computational modeling reveals that the gut bacteria *Akkermansia muciniphila* expresses all the genes encoding the necessary enzymes in the shikimate pathway and accounts for a significant portion of the gene expression associated with the shikimate pathway in the human gut microbiome. These data suggest that *A*. *muciniphila* could be responsible for a significant amount of total shikimate produced by human gut microbiome [36]. We have previously identified higher levels of the *Akkermansia* genus in the gut microbiome of glioma patients and a glioma mouse model, compared to controls [44]. Therefore, the elevated CSF levels of shikimate in GBM patients identified in the current study may be influenced by the gut microbiome, in particular *Akkermansia sp.*, suggesting a possible relationship between the gut-brain axis and CSF metabolites. Additionally, recent studies demonstrate unique microbial signatures within cancer types, including GBM [39]. To determine the source of the CSF shikimate identified in this study, future studies could determine whether shikimate-producing bacteria are present in human GBM tissue [39]. In order to detect remnants of shikimate-producing bacteria in the CSF, metagenomic sequencing could be employed, in a manner similar to but more specific than that used clinically in patients with meningitis or encephalitis [58].

### Uridine levels correlate with tumor samples

We found uridine CSF levels to be greater in pre-treatment GBM samples compared to control samples. Uridine is a pyrimidine molecule that is an intermediate in nucleoside synthesis, particularly in the catabolism of L-glutamine to B-alanine (Sup. Material 3C) [62]. Consistent with our results, uridine concentration was found to be higher in tissue samples of GBM, IDH-wildtype tumors compared to controls [25].

### Tumor mutations influence CSF metabolite levels

Previous studies have demonstrated differences in the levels of metabolites based on genetic alterations in tumors. For example, diffuse gliomas have been shown to exhibit distinct tissue metabolite profiles depending on *IDH1* mutation status [55]. Moreover, our previous study revealed higher levels of D-2-hydroxyglutarate in the CSF of patients with IDH-mutant gliomas compared to patients with IDH-wildtype gliomas [17]. In the current study, we have identified novel differences in CSF metabolites between patients with *TP53*-mutant and *TP53*-wildtype GBM. CSF from patients with *TP53*-wildtype GBM contained higher levels of several metabolites including lactate, GABA, choline, carnitine, and carnitine derivates. In contrast, the levels of lactate, GABA, and choline were increased in the CSF of patients with GBM-*PTEN*-mutant, compared to GBM-*PTEN*-wildtype (Fig. 5).

### Carnitine and derived compounds are more abundant in CSF from patients with *TP53*-wildtype GBM

We identified carnitine and carnitine derivatives as key metabolic biomarkers in the CSF of patients with GBM. Carnitine and its acyl derivatives emerged as significant factors in our dataset, with increased abundance in the CSF of GBM patients, particularly those with *TP53*-wildtype status. Carnitine, acylcarnitines 2-methylbutyrylcarnitine, propionylcarnitine, isobutyryl-L-carnitine, and deoxycarnitine were significantly more abundant in *TP53*-wildtype, compared to *TP53*-mutant, GBM patients (Fig. 6). In GBM patients, *TP53* potentially plays an indirect yet critical role in regulating FAO and may subsequently affect carnitine and acylcarnitine levels in CSF. More specifically, *p53-*responsive elements have been identified in the first intron of the carnitine palmitoyltransferase IC (*CPT1C*) gene; studies in cell lines have shown that *p53* can regulate *CPT1C* expression [48]. The effects of increased *CPT1C* expression on tumor progression and FAO activity is well-documented in the literature [11, 57, 60, 63]. While the exact mechanism for reduced levels of carnitine compounds in the CSF of patients with *TP53*-mutant GBM remains to be elucidated, impaired *TP53* regulation of *CPT1C* expression in *TP53*-mutant GBM may be a contributing factor. When the *TP53* mutations occur, FAO is reduced [48], and the tumor cells’ ability to use FAO as an alternative energy production pathway might be affected. This may result in a reduced demand for carnitine in *TP53*-mutant GBM and lower concentrations of carnitine and carnitine derivatives in the CSF. The present findings support this hypothesis, as the relative levels of carnitine, propionylcarnitine, and isobutyryl-L-carnitine were significantly increased in *TP53*-wildtype GBM but were not significantly different from control patients or patients with *TP53*-mutant GBM (Fig. 6).

### MRS biomarkers for *TP53* and *PTEN* mutation status

The metabolites lactate, GABA, and choline are clinically relevant because they are measured in imaging studies through magnetic resonance spectroscopy (MRS) [56]. Some studies show that tumor mutation status can potentially influence MRS results [30]. Our results reveal that the levels of these metabolites can be influenced by the mutations present in the tumor (Fig. 7). Lactate, GABA, and choline were present at significantly lower levels in the CSF of *TP53*-mutant GBM patients when compared to that of *TP53*-wildtype GBM patients (Fig. 7A and 7C). On the contrary, the relative values of each of the three MRS-measurable metabolites were significantly higher in the CSF of *PTEN*-mutant GBM patients compared to that of *PTEN*-wildtype GBM patients (with the exception of lactate, which follows a similar trend) (Fig. 7D and 7F). Absence of p53 has been associated with downregulation of choline levels in human embryonic stem cells [65], suggesting a potential link between choline metabolism and p53.

### *TP53* and the warburg effect

In addition, *TP53* is known to regulate glycolysis and influence the Warburg effect by negatively regulating lactate dehydrogenase [64]. Similarly, it has been proposed that mutant *TP53* can stimulate the Warburg effect in cultured cells [61]. Interestingly, we observed lower lactate levels in the CSF of patients with mutant *TP53*. The relationship between lower lactate levels in CSF and the predicted effects of mutant *TP53* in regulating the Warburg effect is unclear and requires further investigation. Given that the levels of lactate, GABA, and choline levels appear to be influenced by GBM mutations, it may be important to consider tumor mutations when interpreting MRS results. To evaluate this further, it would be necessary to correlate the CSF metabolite findings with MRS imaging and perform receiver-operator curve analysis to detect the sensitivity and specificity of a given cut-off on MRS that reliably predicts *TP53* and *PTEN* mutation status when GBM tissue is not available.

### Metabolites associated with sex

Differences between male and female patients with glioblastoma have been described. For example, the incidence of glioblastoma is 1.6 times higher in men than women [51]. Also, men have worse OS than women. Although differences in the proportion of MGMT promoter methylation between male and female and female with glioblastoma have been proposed [9], it appears that the difference is not statistically significant [16]. In addition, studies have shown differences in the expression of glycolytic genes between males and females, with potential association to overall survival [23]. In our analysis, we identified some CSF metabolites that were significantly different between male and patients, consistent with the idea that physiological and hormonal differences associated with sex can influence the levels of metabolites in CSF in patients with GBM. This is important when trying to identify metabolites that can serve as GBM biomarkers, because it might be required to use different cutoffs for males and females, when using a CSF metabolite as biomarker to identify patients with GBM.

### Metabolites associated with overall survival

We identified four metabolites that showed a statistically significant association with OS (Fig. 8). Increased levels of 2-methylbutyrylcarnitine and aminobutanal were found to be associated with the presence of GBM, and higher levels were associated with worse OS (Figs. 3B and 3D and 8A and 8B). Aminobutanal is involved in the synthesis of the neurotransmitter GABA [27] and GABA metabolism has been linked to GBM survival [4]. Higher levels of acetylcholine in CSF were found to be associated with worse OS (Fig. 8C). Acetylcholine is a well-defined neurotransmitter with a wide variety of roles in the CNS [37]. GBM invasion was found to be significantly enhanced in brain regions with activation of acetylcholine receptors, and it has been suggested that acetylcholine autocrine signaling facilitates GBM invasion through brain tissue [54]. The same study also found that increased acetylcholine receptor expression in GBM correlated with worse OS.

### Limitations

One limitation of this study is that only CSF samples acquired post-treatment have tumor mutation data available for analysis. Another limitation is the small number of female patients in the post-treatment group. Although, the lower proportion of female patients in the post-treatment GBM group is a limitation, comparison of only the male GBM patients before and after treatment showed significant differences in CSF metabolites. This supports the result that there are differences between the pre- and post-treatment GBM samples that are not due to differences in the number of female patients between the groups. Additional studies with a larger number of patients, including CSF samples acquired before therapy, will provide further confirmation for some of our results. Also, how the time between treatment and CSF collection influences the levels of CSF metabolites is unknown. Despite these limitations, the results of this study increase our understanding of CSF metabolites in GBM patients.

## Conclusion

This study demonstrates differences in CSF metabolites between GBM patients and controls. In particular, the elevated levels of carnitine and 2-methylbutyrylcarnitine point to the role of lipid metabolism in GBM biology. Moreover, the elevated CSF shikimate levels suggest a potentially novel link between GBM and the gut microbiome. In addition, the findings highlight the influence of tumor mutations on CSF metabolite levels, including those that can be independently assessed with non-invasive magnetic resonance spectroscopy.

### Electronic supplementary material

Below is the link to the electronic supplementary material.

**Supplementary Material 1:** Swimmer plot illustrating a simplified survival and event timeline of GBM patients. Events include surgery, chemotherapy, radiation treatment (RT), progression, last follow-up, death, and CSF collection. Some events were omitted or combined for clarity. All available dates for known events are included in Supplementary Material 4. Exact dates for administration of chemotherapy and radiation are not available for patients 29 and 37. **(A)** Swimmer plot of GBM patients who had CSF collected prior to treatment. **(B)** Swimmer plot of GBM patients who had CSF collected after initial treatment

**Supplementary Material 2:** Volcano plots for analysis of metabolic differences based on sex in the GBM cohort. Colored points represent metabolites that are present at significantly different levels (-log10(p-value) > 1.3, log2(Group 1/Group 2) > + 1 or < -1). P-values and fold change calculations for all three figures are available in Supplementary Material 5. **(A)** Comparison of metabolites in the CSF of GBM patients between male and female cohorts. Six CSF metabolites are significantly different between male and female GBM patients. **(B)** Comparison of metabolites in the CSF of pre-treatment GBM patients between male and female cohorts. Four CSF metabolites are significantly different between male and female pre-treatment GBM patients. **(C)** Comparison of metabolites in the CSF of male GBM patients between the pre-treatment and post-treatment cohorts. 12 CSF metabolites are significantly different between pre-treatment and post-treatment male GBM patients

**Supplementary Material 3:** Metabolic pathways of differentially abundant metabolites between pre-treatment GBM and control samples. **(A)** Carnitine shuttle pathway in fatty acid oxidation (FAO). **(B)** Shikimate pathway used by plants, bacteria, algae, and other microorganisms in the biosynthesis of aromatic compounds. **(C)** Catabolism of L-glutamine to B-alanine using uridine as an intermediate. **(D)** Butanoate metabolism with N-acetylputrescine and aminobutanal as intermediate compounds. **(E)** Cholesterol synthesis pathway with farnesyl diphosphate as an intermediate compound

**Supplementary Material 4:** Data used in the study, including clinical data and metabolite levels for all patient samples; treatment and disease progression dates for the GBM cohort; survival data; mutation data for the most common mutated genes; and mutation frequencies for all mutated genes

**Supplementary Material 5:** Significance values and fold change values for: metabolites that are significantly different and meet the fold change cutoff (+/-1) between pre-treatment and post-treatment GBM samples; all measured CSF metabolites between male and female patients in the GBM cohort; all measured CSF metabolites between male and female patients in the pre-treatment GBM cohort; and all measured CSF metabolites between pre-treatment and post-treatment samples in the GBM-male-only cohort

**Supplementary Material 6:** Methods for metabolomic analysis of CSF samples

## Data Availability

All data generated or analyzed during this study are included in this published article and its supplementary information files.
