# Supplementary figures and images for: The genomic alterations in glioblastoma influence the levels of CSF metabolites
Source: Acta Neuropathol Commun. 2024 Jan 19;12:13. doi: 10.1186/s40478-024-01722-1 (PMC10799404; doi:10.1186/s40478-024-01722-1)

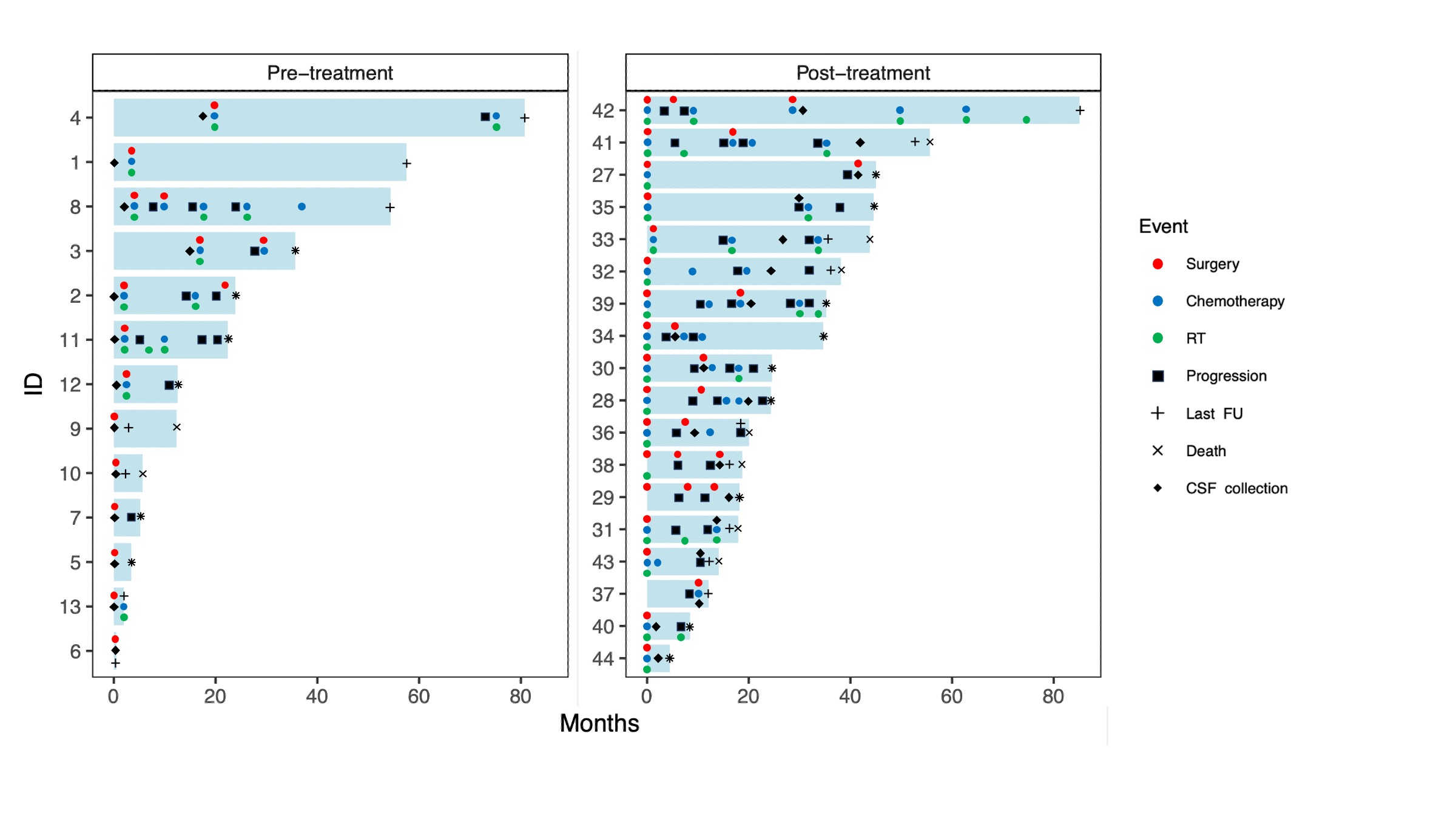

Supplement: Supplementary file 1 — Supplementary Material 1: Swimmer plot illustrating a simplified survival and event timeline of GBM patients. Events include surgery, chemotherapy, radiation treatment (RT), progression, last follow-up, death, and CSF collection. Some events were omitted or combined for clarity. All available dates for known events are included in Supplementary Material 4. Exact dates for administration of chemotherapy and radiation are not available for patients 29 and 37. (A) Swimmer plot of GBM patients who had CSF collected prior to treatment. (B) Swimmer plot of GBM patients who had CSF collected after initial treatment [file 40478_2024_1722_MOESM1_ESM.jpeg]

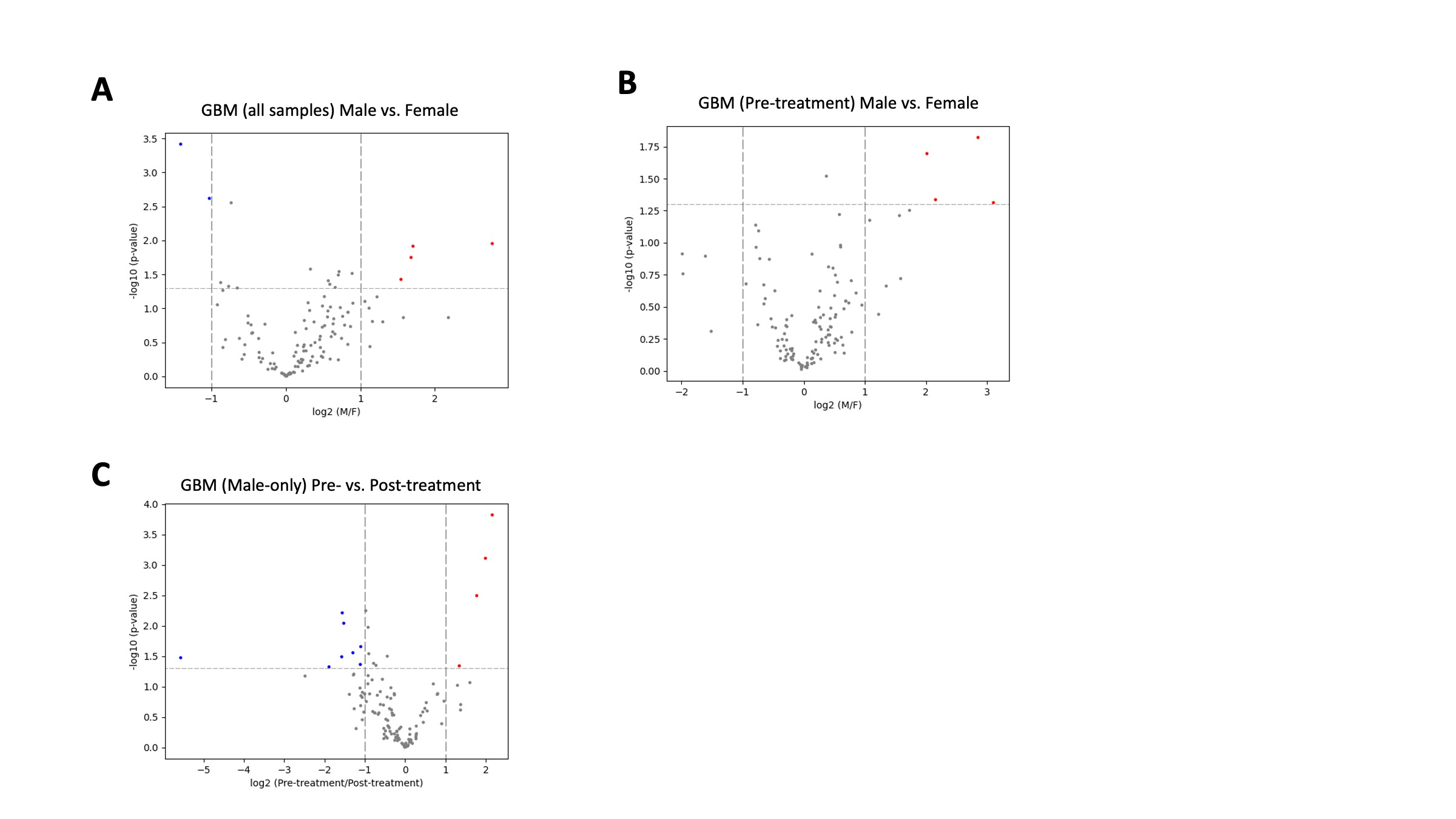

Supplement: Supplementary file 2 — Supplementary Material 2: Volcano plots for analysis of metabolic differences based on sex in the GBM cohort. Colored points represent metabolites that are present at significantly different levels (-log10(p-value) > 1.3, log2(Group 1/Group 2) > + 1 or < -1). P-values and fold change calculations for all three figures are available in Supplementary Material 5. (A) Comparison of metabolites in the CSF of GBM patients between male and female cohorts. Six CSF metabolites are significantly different between male and female GBM patients. (B) Comparison of metabolites in the CSF of pre-treatment GBM patients between male and female cohorts. Four CSF metabolites are significantly different between male and female pre-treatment GBM patients. (C) Comparison of metabolites in the CSF of male GBM patients between the pre-treatment and post-treatment cohorts. 12 CSF metabolites are significantly different between pre-treatment and post-treatment male GBM patients [file 40478_2024_1722_MOESM2_ESM.jpeg]

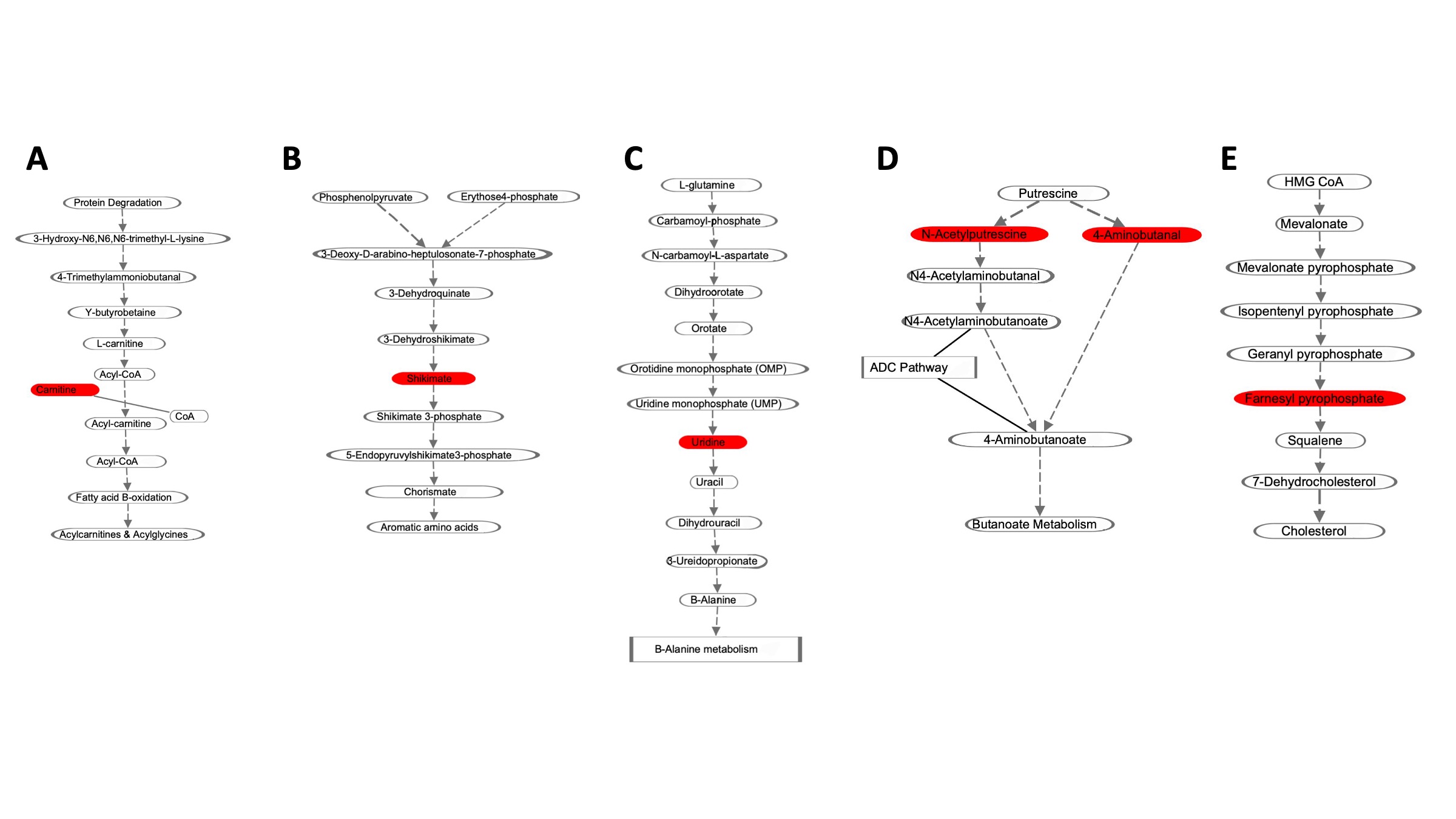

Supplement: Supplementary file 3 — Supplementary Material 3: Metabolic pathways of differentially abundant metabolites between pre-treatment GBM and control samples. (A) Carnitine shuttle pathway in fatty acid oxidation (FAO). (B) Shikimate pathway used by plants, bacteria, algae, and other microorganisms in the biosynthesis of aromatic compounds. (C) Catabolism of L-glutamine to B-alanine using uridine as an intermediate. (D) Butanoate metabolism with N-acetylputrescine and aminobutanal as intermediate compounds. (E) Cholesterol synthesis pathway with farnesyl diphosphate as an intermediate compound [file 40478_2024_1722_MOESM3_ESM.jpeg]
