# Supplementary material for: The genomic alterations in glioblastoma influence the levels of CSF metabolites
Source: Acta Neuropathol Commun. 2024 Jan 19;12:13. doi: 10.1186/s40478-024-01722-1 (PMC10799404; doi:10.1186/s40478-024-01722-1)
Supplement: Supplementary file 6 — Supplementary Material 6: Methods for metabolomic analysis of CSF samples [file 40478_2024_1722_MOESM6_ESM.docx]

**Supplementary methods**

Metabolites extraction from CSF involved using a CSF pool for quality control after the extraction process. All CSF samples for the study were stored at -80 °C. The metabolic extraction used 100 µL of CSF. The extraction procedure was described earlier publications from our group (PMID: 32868820). Following the extraction samples were dried and re-suspended 1:1 methanol: water. The extract was deproteinized using a 3 kDa molecular filter (Amicon ultracel-3K Membrane; Millipore Corporation, Billerica, MA) and the filtrate was dried under vacuum (Genevac EZ-2plus; Gardiner, Stone Ridge, NY). Prior to mass spectrometry (MS), the dried extracts were re-suspended in identical volumes of injection solvent composed of water:methanol (1:1) and were subjected to liquid chromatography-MS (LC-MS). LC-MS-extracted CSF samples were injected and analyzed using a 6490 triple quadrupole mass spectrometer (Agilent Technologies, Santa Clara, CA) coupled to a HPLC system (Agilent Technologies, Santa Clara, CA) via single reaction monitoring (SRM). Source parameters were as follows: gas temperature 250 °C; gas flow 14 L/min; nebulizer 20 psi; sheath gas temperature 350 °C; sheath gas flow 12 L/min; capillary 3,000 V positive and 3,000 V negative; nozzle voltage 1,500 V positive and 1,500 V negative. Approximately 8-11 data points were acquired per detected metabolite. We used three different analytical methods to measure the metabolites as described earlier publications [1].

REFERENCES

1 Takayasu T, Shah M, Dono A, Yan Y, Borkar R, Putluri N, Zhu JJ, Hama S, Yamasaki F, Tahara Het al (2020) Cerebrospinal fluid ctDNA and metabolites are informative biomarkers for the evaluation of CNS germ cell tumors. Sci Rep 10: 14326 Doi 10.1038/s41598-020-71161-0
